# Development a stacking pad design for enhancing the sensitivity of lateral flow immunoassay

**DOI:** 10.1038/s41598-018-35694-9

**Published:** 2018-11-23

**Authors:** Tsung-Ting Tsai, Tse-Hao Huang, Chung-An Chen, Natalie Yi-Ju Ho, Yi-Ju Chou, Chien-Fu Chen

**Affiliations:** 1 Department of Orthopaedic Surgery, Bone and Joint Research Center, Chang Gung Memorial Hospital and Chang Gung University College of Medicine, Taoyuan, 333 Taiwan; 2 Institute of Applied Mechanics, National Taiwan University, Taipei, 106 Taiwan

## Abstract

Lateral flow immunoassays (LFIAs) have wide application in point-of-care testing, particularly in resource-poor settings. To achieve signal amplification in a gold nanoparticle-based lateral flow assay without an additional procedure or the need for complex fabrication, a new and simple method was developed for using a “stacking pad” configuration that adds an additional membrane between the conjugation pad and test pad to the conventional AuNP-based LFIA format. This design helps to accumulate the antibody and antigen on the stacking pad, hence extending the antigen/antibody binding interactions to enhance the test’s detection sensitivity. With the enhanced lateral flow assay, as low as 1 ng/mL of Protein A and 15.5 ng/mL of C-reactive protein can be visualized with the naked eye. We also successfully applied the stacking pad system in the analysis of C-reactive protein in human serum and synovial fluid samples. These results suggest that this stacking pad LFIA can provide sensitive and on-site prognosis for detection in synovial fluid and serum samples in resource-limited settings.

## Introduction

A lateral flow immunoassay (LFIA) is a simple diagnostic device based on the chromatography-like migration of a labeled analyte through multiple membranes, including a sample pad, conjugation pad, detection pad, and absorbance pad, ending in the visible result of an immobilized captured reagent. The sample pad ensures the controlled flow of the test solution, which migrates to the conjugate pad where nanoparticles labeled with antibodies are stored. If the target analyte is present, the labeled antibodies will bind to it and continue to migrate to the detection pad, whereupon the materials are captured by immobilized antibodies at a test line (T-line) to form a coloured strip while a subsequent control line (C-line) is used to colorimetrically indicate that the solution has sufficiently migrated. Finally, the absorbent pad absorbs excess sample. The test solutions can be driven by capillary force through the porous network of the fibrous pads without the use of an external pump, enabling simple confirmation of the presence or absence of a target analyte by visually observing the signal intensity at the T- and C-lines. Due to this simplicity, LFIAs have been used in a variety of settings, including clinical, food safety, and environmental analyses. Compared to standard laboratory technologies, LFIAs are simple-to-use, rapid, low-cost, and portable, thus meeting the criteria for healthcare in resource-limited settings^1,2^, and have been widely used for the detection of various targets, such as tumor markers^3,4^, bacterium^5^, viruses^6^, nucleic acids^7^, and pesticide residues^8^. However, poor sensitivity limits the further application of this testing platform^9^.

While gold nanoparticles (AuNPs) are the most common material conjugated with antibodies for colorimetric signaling purposes, one way of improving the detection sensitivity of a AuNP-based LFIA is by enhancing the readout signal with various amplification strategies. A simple and effective way of doing so involves the so-called silver enhancement technique^10–12^, in which AuNPs are used as nuclei under a reducing environment for the deposition of metallic silver in order to amplify electrochemical signals. Although silver enhancement is widely used to improve AuNP-based LFIA systems, the reagents are relatively unstable and highly light sensitive. Another approach to enhance the assay sensitivity is by immobilizing and enabling the enzymatic activity of horseradish peroxidase or alkaline phosphatase on the surface of AuNPs to catalyze the conversion of chromogenic substrates (e.g., 3,3′,5,5′-tetramethylbenzidine, p-nitrophenyl phosphate disodium salt, and 2,2′-azinobis [3-ethylbenzothiazoline-6-sulfonic acid]-diammonium salt) into darker colored products than AuNPs alone^13^. However, the need to immobilize and store the enzymatic reagents at low temperature may hinder the platform for use in resource-limited environments. Dual AuNP conjugate-based lateral flow assays have also been reported that are based on the surface plasmon resonance effect of the AuNP-antibody conjugates for signal amplification^14–16^. However, cumbersome processing and longer incubation times are required to perform these tests.

Other research groups have worked to improve the sensitivity of LFIAs by modifying the testing platform’s architecture. For example, a dialysis-based concentration method integrated with an LFIA device has been developed for low concentration targets^17^, and two- and three-dimensional paper networks that are capable of multiple tasks, such as multiplexing, sample processing, and signal enhancement, have been embedded in different layers of the membranes that compose the platform^18–20^. Moreover, different types of geometries have been developed to manipulate the fluidic flow and to retain operational simplicity^21–23^. However, the need for complex fabrication steps that limit scaling ultimately restrict the practical applications of such tests. Developing a one-step manually operated device that affords sequential delivery of multiple fluids for analyte detection remains a challenge.

In this paper, we introduce an innovative “stacking pad” configuration by adding an additional membrane between the conjugation pad and test pad to the conventional AuNP-based LFIA format (sLFIA), which can accumulate the antibody and antigen on the stacking pad, hence extending the antigen/antibody binding interactions to enhance the test’s detection sensitivity. This concept was adapted based on the function of the stacking gel in polyacrylamide gel electrophoresis (PAGE), which allows for proteins to be packed in a concentrated area, thus enabling increased antibody/antigen interaction time^24–26^. In this work, the incorporation of a similar “stacking pad” in a membrane-based platform was demonstrated to extend the binding interaction of antigens and antibodies. In addition, various membrane materials, including polyester, cellulose, and glass fiber, were examined as the stacking pad to further increase the detection limit of the colorimetric signal.

In order to verify the feasibility of the proposed platform, we chose Protein A, a product of *Staphylococcus aureus*, as a targeted biomarker^27^. *S*. *aureus* is one of the most important human pathogens and a major cause of surgically implanted device-related infection frequently responsible for prosthetic joint infection (PJI)^28–32^, which remains a major clinical issue that resists even aggressive treatment by potent antibiotics^33^. The standard protocol of *S*. *aureus* diagnosis requires culturing of an isolated *S*. *aureus* on a blood agar plate and then a latex test to identify the bacteria—a process that is both time- and resource-intensive. A better detection strategy involves the targeting of a characteristic protein of the bacteria. In this case, Protein A is a constituent of the cell wall of *S*. *aureus* and can be utilized as a targeted analyte. Additionally, for diagnosis of infection, serum C-reactive protein (CRP) is a simple and inexpensive test that is now widely used^34^. CRP is reflective of systemic changes in infection, and its level is the measurement of acute phase reactants, which can be specifically utilized to improve the specificity of the combined algorithm, given that its reasonably high sensitivity and acceptable specificity for detecting infection^35–37^. To study the ability of our sLFIA platform to detect Protein A and CRP, we measured multiple threshold levels of the molecule in PBS buffer and determined the test can accurately visualize the target with a detection limit of 1 ng/mL and 15.5 ng/mL of Protein A and CRP, respectively, in as little as 20 min. We also successfully validated the performance of our CRP assay in a small trial using the actual synovial fluid and serum samples to simulate the rapid diagnosis of PJI during operation.

## Results

### Protein A detection using the sLFIA

**Figure 1:**
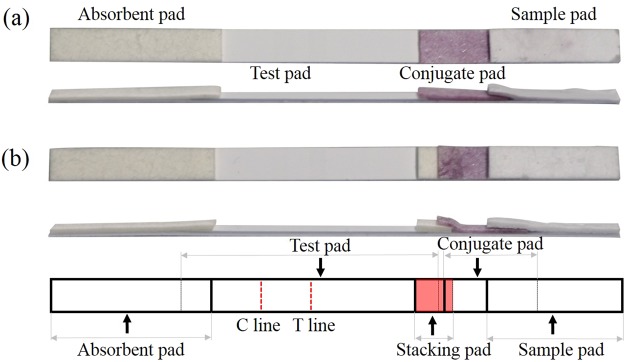
Images and design of the lateral flow assay device. (**a**) The top (upper) and side (bottom) view of a conventional LFIA. The test assembly consists of a sample pad, a conjugation pad, a test pad, and an absorbent pad. (**b**) The assembled sLFIA device that has an additional stacking pad added between the conjugate and test pads.

Compared to the conventional LFIA device (Fig. 1(a)), the proposed sLFIA shown in Fig. 1(b) integrates an additional stacking pad between a detection pad that dry-stores the capture and control probes, and a conjugate pad that dry-stores the AuNP-anti-Protein A or AuNP-anti-CRP conjugates using 30 nm AuNPs. The stacking pad configuration was adapted to mimic PAGE stacking gels, which allows for longer sample interaction with the AuNP-anti-Protein A or AuNP-anti-CRP conjugates. The test pad immobilizes the anti-Protein A or anti-CRP molecules and secondary antibodies at the T- and C-lines for capturing Protein A or CRP bound AuNP-anti-Protein A/CRP conjugates and AuNP-anti-Protein A/CRP conjugates, respectively. Finally, a cellulose fiber absorbent pad collects the waste sample at the end of the device. All parts were assembled according to Fig. 1(b).

**Figure 2:**
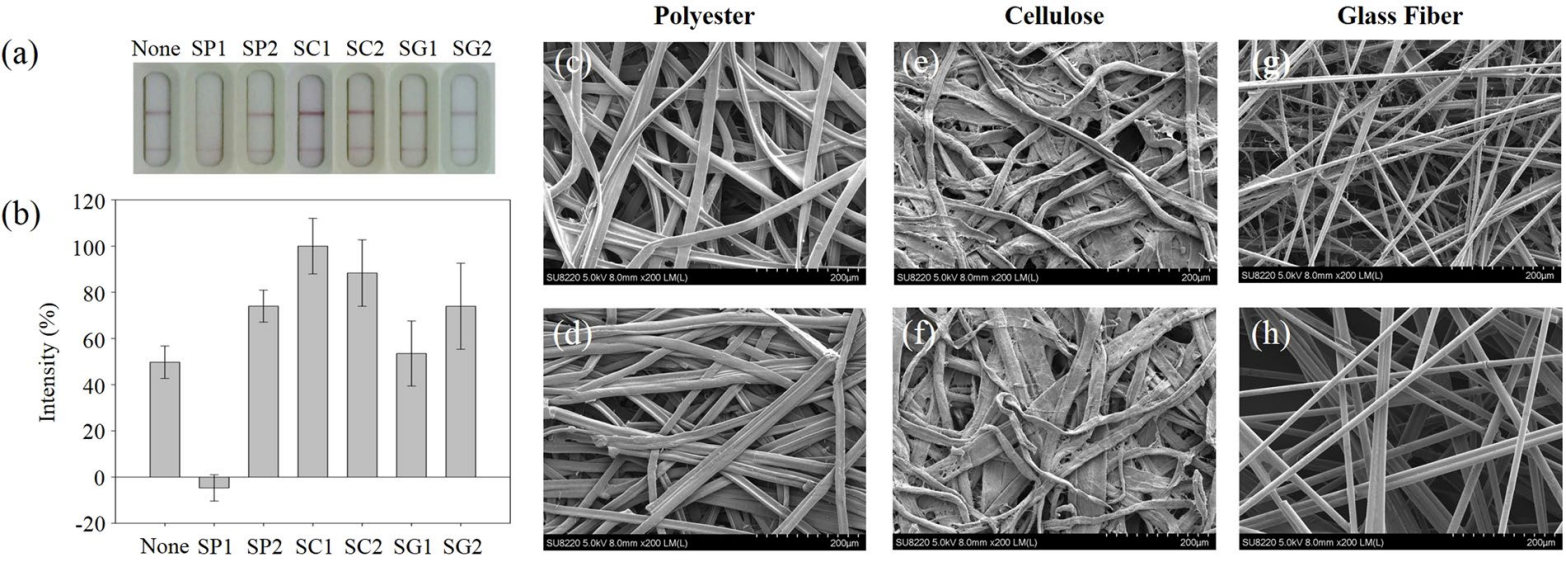
Comparison of various stacking pad materials used in the sLFIA test. (**a**) Photograph of the test results using different stacking pads. (**b**) Plot of the normalized colorimetric signal intensity of 10 ng/mL concentrated Protein A using various stacking pads.“None” corresponds to the conventional LFIA. SEM images of the different membranes employed as stacking pads in the sLFIA test: (**c**) SP1 (0.5 mm thick); (**d**) SP2 (0.1 mm thick); (**e**) SC1 (0.5 mm thick); (**f**) SC2 (1 mm thick); (**g**) SG1 (0.6 mm thick); and (h) SG2 (0.2 mm thick).

We tested the effect of various compositions of the stacking pad. Six different materials were tested from three categories, including polyester (SP1 and SP2), cellulose (SC1 and SC2), and glass fiber (SG1 and SG2), to examine their respective performance for Protein A detection. Additionally, to precisely and quasi-quantitatively study the detection performance of the different stacking pad substrates, the signal of the AuNP aggregates on the T- and C-lines were further measured via optical intensity analysis using ImageJ software. Figure 2 shows the optical images and corresponding intensities of the T-line for 10 ng/mL Protein A spiked in PBS buffer tests on the different stacking pad materials. Compare to the similar thickness of SP1 (0.5 mm), SC1 (0.5 mm), and SG1 (0.6 mm), the results demonstrate that the SC1 cellulose substrate displayed the best performance, in which the intensity increased by almost 2-fold compared to the LFIA test without a stacking pad. However, not all materials can be used to enhance the response signal intensity in this system. Based on the results shown in Fig. 2(b), the glass fiber-based membrane SG1 showed comparable performance to the conventional LFIA test, and the polyester-based membrane SP1 stacking pad not only did not enhance the sensing results, but the signal dramatically also decreased at the T-line compared to the LFIA without a stacking pad. This suggests that the AuNP conjugates were absorbed by the SP1 and unable to release to the subsequent test pad with the fluid flow, which causing the diminution of the signal at the T-line.

To examine the effect of the thickness of the stacking pad, two different thicknesses of the same material were used. The test results of the normalized colorimetric signal intensity showed that the thinner cellulose membrane (SC1, 0.5 mm) possessed 1.13-fold enhanced intensity compared to the SC2 which was 1 mm thick (Fig. 2(b)). In addition, thinner membranes of polyester (SP2, 0.1 mm) and glass fiber (SG2, 0.2 mm) also displayed 70- and 1.38-fold enhanced intensity compared to thicker membranes of polyester (SP1, 0.5 mm) and glass fiber (SG1, 0.6 mm), respectively. These results can be attributed to the fact that SC2, SP1, and SG1 have larger capacity than SC1, SP2, and SG2 to accommodate the sample fluid; however, this may create a lower density of AuNP-anti-Protein A conjugates distributed throughout the membrane network. While the sample fluid flows through the conjugation and stacking pads, a larger amount of antibody-AuNPs-Protein A complexes can become trapped before they can be captured at the T-line.

### Characterization of the different stacking pad materials

To investigate the effect of the stacking pad material, we characterized the morphology of these substrates using SEM (Fig. 2). The results showed that the cellulose-based stacking pads possessed the tightest fiber packing (Fig. 2(e,f)), followed by the polyester-based (Fig. 2(c,d)), while the glass fiber substrates showed the loosest packing (Fig. 2(g,h)). The compact nature of the cellulose-based stacking pads (Fig. 2(e,f)), with small pore sizes in the scaffold results in the lowest permeability, which may contribute to the slower flow rate of the Protein A spiked in PBS buffer solution, thus increasing the duration of the Protein A and AuNP-anti-Protein A binding reaction by diffusion at the conjugate pad. In addition, the stacking effect and the longer interaction time caused by the smaller pores in the cellulose stacking pads allows a much higher amount of target analyte to interact with the antibody at the conjugation pad, so signal enhancement of the test results is observed.

Previous studies have also reported that the material of the stacking pad is an important parameter for lateral flow assays. The architecture and design of the selected paper can cause signal changes of the optical sensing system^38^. Due to the good protein adsorption of polyester films, the analytes and probes remained on the stacking pad and the intensity on the nitrocellulose membrane was dramatically decreased (Fig. 2b)^39^. Moreover, nonspecific adsorption may occur on the surface of glass fiber with trace metal ions to slightly lower the signal intensity^40^. These results indicate that cellulose has superior surface property compared to polyester and glass fiber. Next, the effect of paper thickness was also examined using different materials for this lateral flow system. As the thickness of stacking pads increases in this analytical system, the diffusion of gold nanoparticles leads to low-density probes on the pads, which results in a decreased signal intensity^38^. Based on the findings of previous literatures and our study, the moderate thickness of 0.5 mm cellulose (SC1) for a stacking pad was selected.

**Figure 3:**
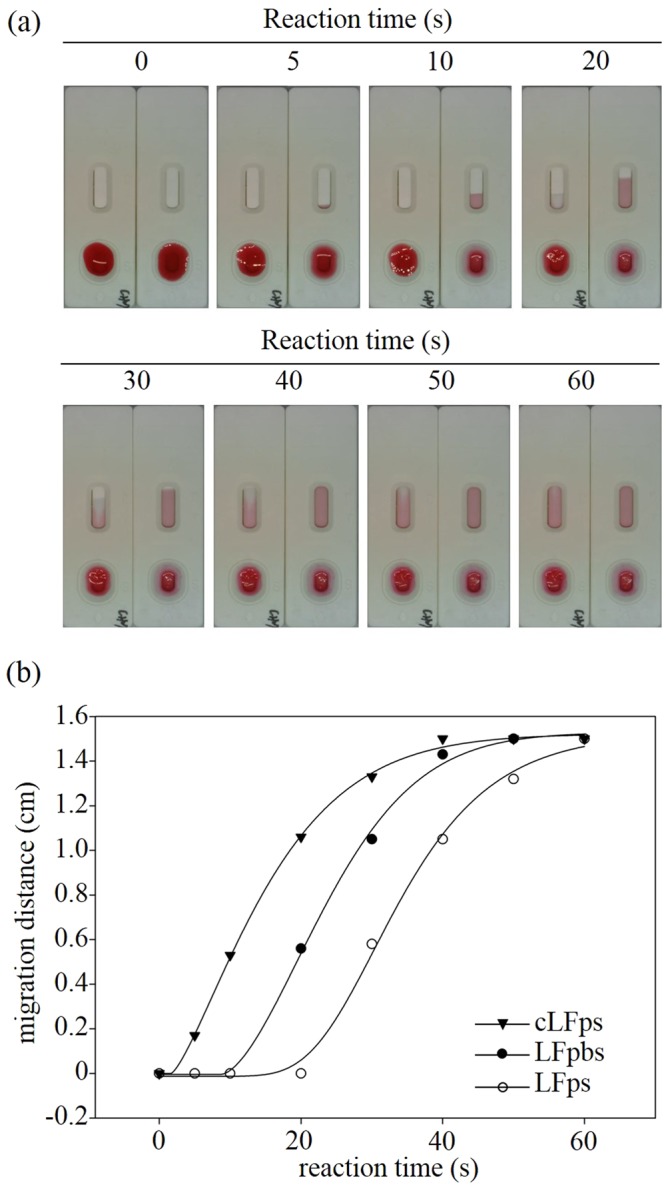
Migration distance as a function of time with and without the stacking pad. (**a**) Images of various reaction times with (left) and without (right) the stacking pad. (**b**) The scatter plot shows the migration distance of the liquid front of the conventional LFIA device featuring no stacking effect (cLFps), and the PBS liquid front position (LFpbs) and the Ponceau S liquid front position (LFps) of the sLFIA device as measured from the front of the reading window to the end of the reading window to assess the stacking effect.

**Figure 4:**
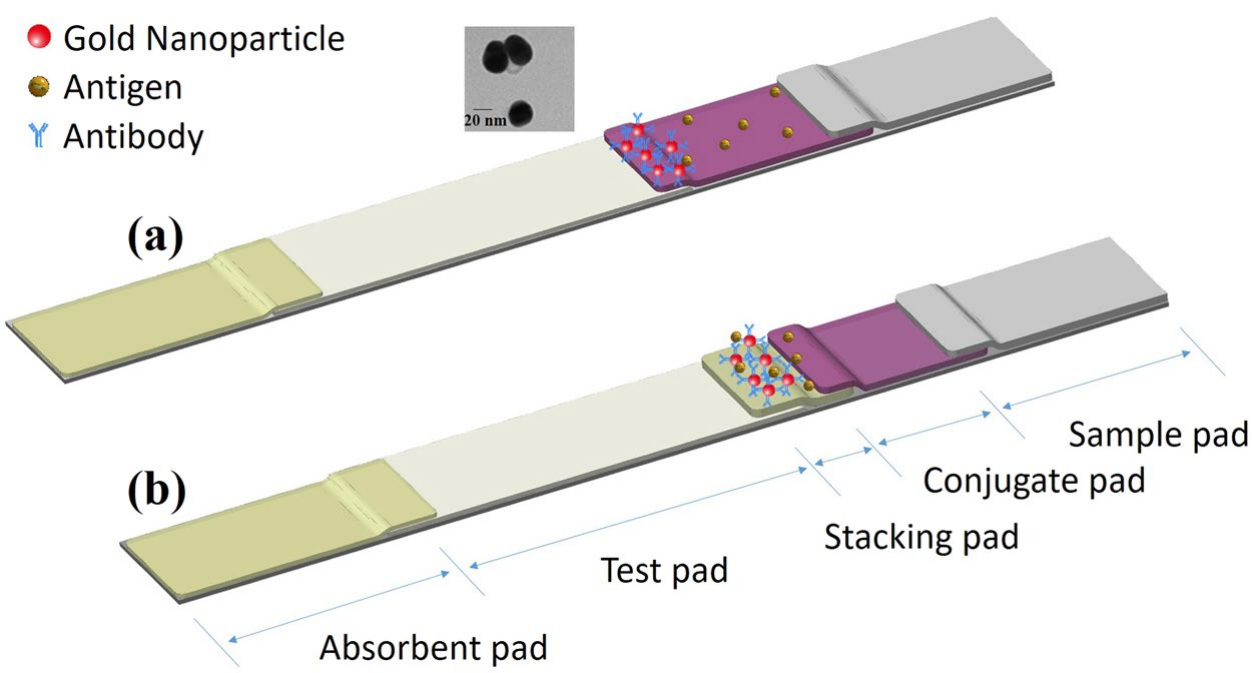
Proposed mechanism of the LFIA and sLFIA tests. (**a**) In the conventional LFIA test, the sample fluid simply flows through the conjugate pad and directly enters the test pad where the antigens behind the sample fluid do not contact the antibodies on the conjugate pad. (**b**) In the stacking effect of the sLFIA device, the sample fluid dissolves the AuNP-anti-Protein A conjugates and enters the stacking pad due to the smaller pores of the scaffold, concentrating the AuNP-anti-Protein A conjugates and antigens in the stacking pad, and thus increasing the binding time and interaction probability. The insert is the TEM image of 30 nm AuNPs.

In addition, we also observed the liquid flow of the added sample solution passing through all the membranes by applying 1/100 diluted red Ponceau S dye solution to the sample pad to illustrate the stacking effect (Fig. 3). Fluid flow within a capillary network is driven by capillary pressure, *Pc* = 2*γ*cos*θ*/*R*, in which *γ* is the surface tension of the fluid, *R* is the capillary radius, and *θ* is the contact angle of the liquid-air interface with the solid. Figure 3(a) displays various reaction times, in which the Ponceau S solution entered the observation window after 5 s for the convention LFIA without the stacking effect, while the flow time extended to approximately 15 s using the stacking pad in the sLFIA device. The data shows that using the stacking pad results in longer migration time. However, the clear PBS buffer liquid front of the sample fluid enters the reading window approximately 7 s in advance of the Ponceau S stain in the sLFIA device (Fig. 3(b)). This demonstrates that the sample fluid can be efficiently held and concentrated at the interface between the conjugate pad and the test pad by the stacking pad, thus extending the reaction time and concentration of the Protein A and AuNP-anti-Protein A conjugates on the stacking pad to increase the binding opportunity. A schematic illustration of the flow field is shown in Fig. 4. Figure 4(a) displays the conventional LFIA, in which the sample fluid simply flows through the conjugate pad and directly enters the test pad where the antigens behind the sample fluid do not contact the antibodies on the conjugate pad. Meanwhile, the stacking effect of the sLFIA device is shown in Fig. 4(b), in which the sample fluid dissolves the AuNP-anti-Protein A conjugates and enters the stacking pad due to the smaller pores of the scaffold, concentrating the AuNP-anti-Protein A conjugates and antigens in the stacking pad, and thus increasing the binding time and probability.

### Quantification of Protein A and CRP

**Figure 5:**
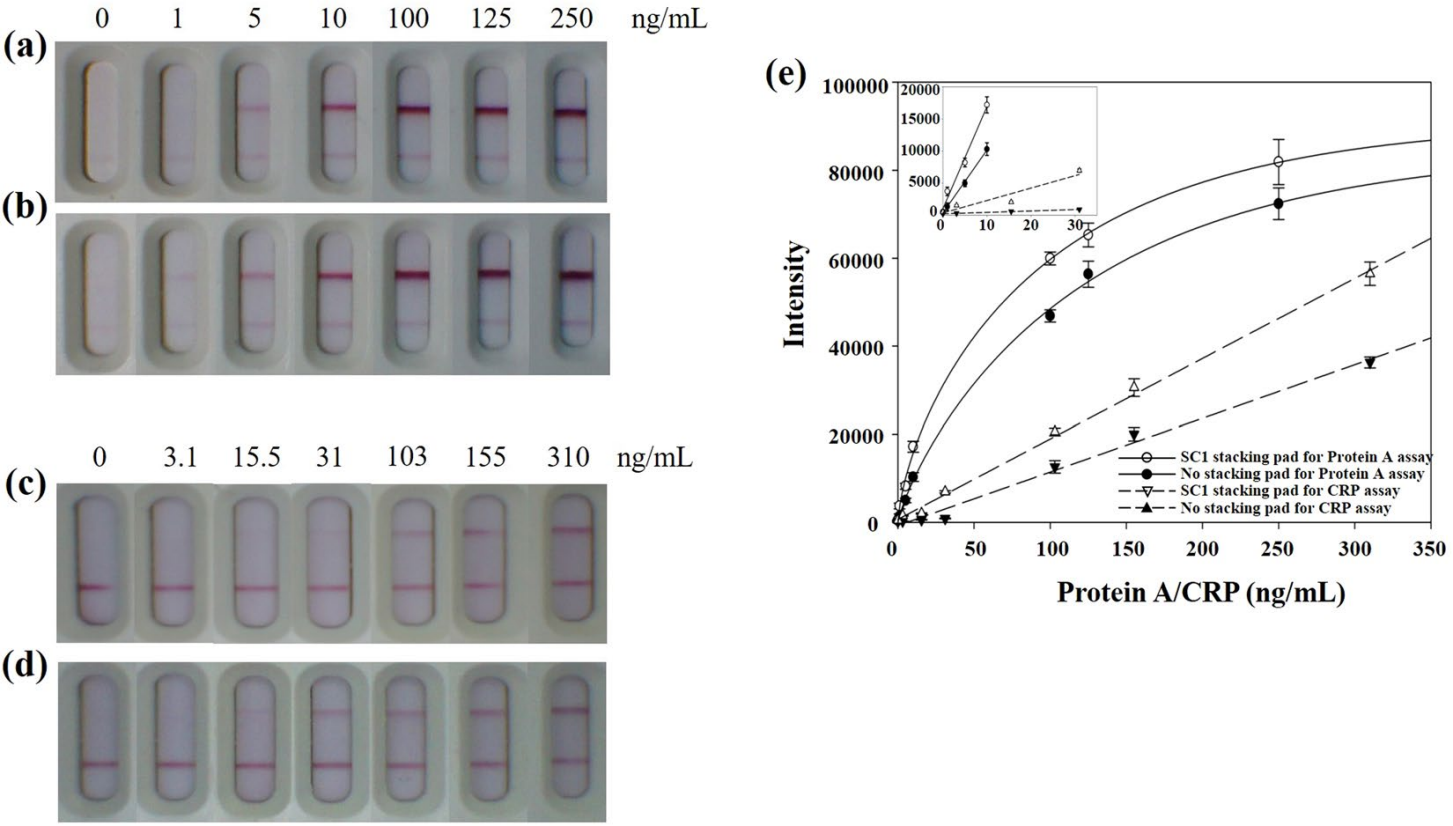
Photographs of test strips showing Protein A spiked PBS samples by (**a**) the conventional LFIA and (**b**) sLFIA (with SC1 stacking pad). Photographs of test strips showing CRP spiked PBS samples by (**c**) the conventional LFIA, and (**d**) sLFIA (with SC1 stacking pad). (**e**) Plot of various Protein A and CRP concentrations spiked in PBS buffer. The error bars represent the standard deviation of three independent experiments.

To evaluate the practical use of the sLFIA device, we chose Protein A, a product of *S*. *aureus*, and CRP, whose production increases in response to inflammation as the target molecules. First, Protein A spiked PBS solution (0–250 ng/mL) and CRP spiked PBS solution (0–310 ng/mL) were chosen as the sample solutions to reduce the matrix effect. As shown in Fig. 5, the intensity of the red lines in the T-line increased with increasing concentrations of Protein A and CRP. The detection signal was significantly enhanced by our stacking pad system, in which Protein A and CRP were visible even as low as 1 ng/mL (Fig. 5(b)) and 15.5 ng/mL (Fig. 5(d)), respectively, when compared to 5 ng/mL (Fig. 5(a)) and 31 ng/mL (Fig. 5(c)) by the conventional LFIA method. In the absence of Protein A or CRP, no red band was observed at the T-line. The resulting calibration curve is shown in Fig. 5(e). Therefore, the signal enhancement method developed in this study was 5- and 2-times more sensitive than the normal LFIA test for the detection of Protein A and CRP, respectively (the inset of Fig. 5(e)).

**Figure 6:**
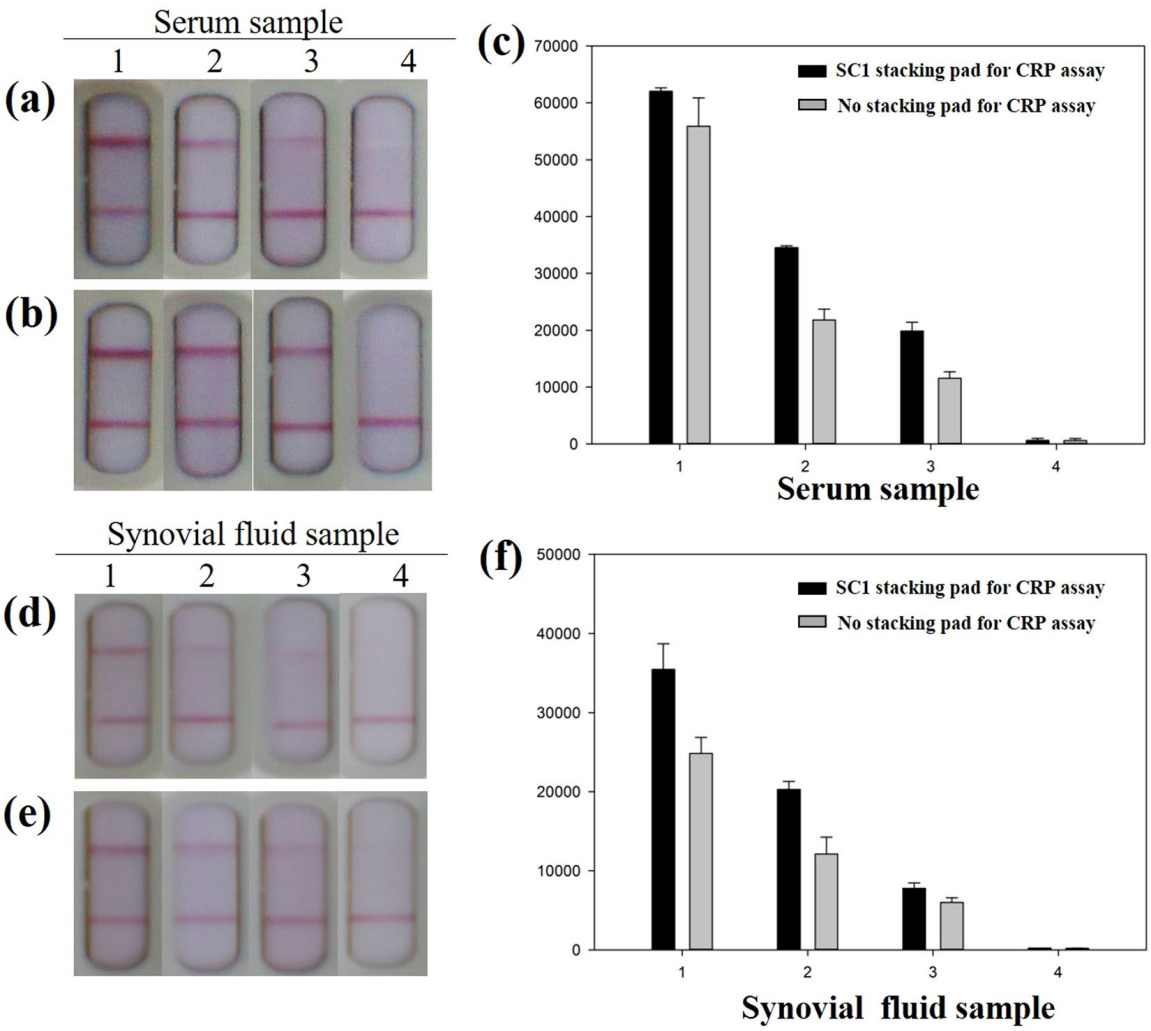
Photographs of test strips showing human serum samples of various CRP concentrations by (**a**) the conventional LFIA and (**b**) sLFIA (with SC1 stacking pad). (**c**) The optical intensity of serum samples analyzed using ImageJ. Photographs of test strips showing human synovial fluid samples of various CRP concentrations by (**d**) the conventional LFIA and (**e**) sLFIA (with SC1 stacking pad). (**f**) The optical intensity of synovial fluid samples analyzed using ImageJ. The error bars represent the standard deviation of three independent experiments.

To explore the possibility of using the sLFIA platform for clinical tests, two different sample matrices of various CRP concentrations from high (~99.5 mg/L) to low (<1 mg/L) in human serum and synovial fluid samples were used to examine the accuracy of tests in these complex mediums. As shown in Fig. 6, the signals were visually detected in the 1/100 diluted serum and synovial fluid samples. According to the cut-off value of CRP in normal, healthy subjects, CRP levels are usually less than 5 mg/L^41–44^, the CRP levels in serum samples were divided into three groups for evaluating the performance of sLFIA. The results of test strips by conventional LFIA (Fig. 6(a)) and sLFIA (Fig. 6(b)) for four serum samples with CRP level of high (99.45 mg/L), medium (10.34 mg/L and 5.37 mg/L) and low (<1 mg/L) showed that the optical intensity of CRP assay was correlated with the concentration of each serum sample, which were analyzed by ImageJ (Fig. 6(c)). As expected, the optical intensities of our sFLIA device were higher than those by the conventional LFA by 1.1- to 1.7-fold, where there’s no signal detected under serum CRP cut-off value.

Moreover, the serum level of CRP has also long been considered as a useful parameter for musculoskeletal and prosthetic joint infections. Some evidence suggests that measuring CRP in synovial fluid can improve its accuracy^45^, with proposed cutoff values ranging from 1.8 mg/L to 9.5 mg/L^36,46–48^. The performance of sLFIA was further examined by three levels of CRP in human synovial fluid samples. The results of test strips by conventional LFIA and sLFIA are shown in Fig. 6(d,e) respectively, and the optical intensity of synovial fluid samples on the CRP assay is shown in Fig. 6(f), with CRP level of high (10.8 mg/L), medium (5.6 mg/L and 2.7 mg/L) and low (<1 mg/L). Similar to the results of human serum samples, the optical intensities were also correlated with the concentration of each synovial fluid sample, and the optical intensities of our sLFIA device were higher than those by the conventional LFA by 1.3- to 1.7-fold.

Furthermore, the recoveries of CRP are summarized in Table 1. The equation of linear regression for the calibration curve of the CRP spiked in PBS buffer solution is y = 182.24x + 922.95 with R^2^ = 0.9965, in the concentration range of 15.5–310 ng/mL, where Y and X indicate the intensity and concentration, respectively. Recoveries were calculated by the comparison between different CRP levels measured by Hitachi LST008 and the intensity obtained from spiked CRP assay in human serum and synovial fluid samples. In the case of low concentration samples, CRP was undetected thus the recovery was not analyzed. The recovery of CRP in serum samples ranged from 34% to 193%, where the intensity of high CRP level (994.5 ng/mL) may be saturated, and the concentration was underestimated. Therefore, for high-level CRP assay in serum, sLFIA was only 1.1-fold more enhanced to conventional LFIA. The recovery of CRP in synovial fluid sample ranged from 139% to 189%, where the recoveries of medium CRP level (50~100 ng/mL) were similar for both samples. The results indicate that the increased matrix effect and viscosity can extend the duration of the flow time, which can increase the binding time of CRP and AuNP-anti-CRP, thus enhance the optical intensity. Our sLFIA device once again proved to be satisfactory for detection of these kinds of samples. The enhanced-sensitivity of the sLFIA device could potentially be used to quickly diagnose or reassure an individual about their infection status, and the simple design could be widely used as a clinical pre-screening kit.

**Table 1:** Recovery of CRP in human serum and synovial fluid samples.

Sample	Measured concentration (ng/mL)	Observed by sLFIA (ng/mL)	Recovery (%)
Serum (1/100x dilution)	994.5	335.4	34
	103.4	183.9	178
	53.7	103.6	193
	<10	<10	n.a.
Synovial fluid (1/100x dilution)	108	188.9	175
	56	105.9	189
	27	37.5	139
	<10	<10	n.a.

n.a.: not analyzed.

## Discussion

In this work, we developed an enhanced-sensitivity LFIA method for the detection of Protein A (characteristic of *S*. *aureus*) and CRP without the need for additional operational steps to amplify the signal. An additional stacking pad was introduced to a conventional AuNP-based LFIA system, which we demonstrated can enhance the detection signal by 2-fold compared to the conventional LFIA test, with a visible sensitivity of 1 ng/mL and 15.5 ng/mL of Protein A and CRP, respectively. The sLFIA test can also be used to successfully enhance the intensity of CRP assay in human serum and synovial fluid samples by 1.2- to 1.7-fold. Not only does the stacking pad extend the interaction time but also increases the interaction probability between the target analyte and AuNP-labeled antibodies, thus enhancing the test performance. In addition to serum and synovial fluid samples, the stacking pad can be widely applied to other types of sample matrices, such as urine, and sweat, which feature lower viscosity than synovial fluid. Additionally, various signal enhancement strategies, such as adding silver enhancement to the AuNPs or using fluorescent nanomaterials as the signal labels of detection antibodies, can be further adopted to enhance the detection limit of the platform^49,50^.

## Methods

### Reagents and equipment

Methanol (99%), 2-propanol (99%), ethanol (99%), trisodium citrate (>99%), hydrogen tetrachloroaurate (III) trihydrate (99%), borate buffer, sucrose, Ponceau-S stain, polyclonal anti-Protein A antibody, recombinant Protein A and CRP were purchased from Sigma-Aldrich (St. Louis, MO, USA). Anti-CRP antibody was purchased from Arista Biologicals Inc. (Allentown, PA, USA). Goat anti-mouse immunoglobulin (IgG) antibody was supplied by Jackson ImmunoResearch Laboratories (West Grove, PA, USA). We used ultrapure water (18.2 mΩ·cm) throughout the experiments, which was filtered through a Milli-Q system (Millipore, Milford, MA). Test pad (nitrocellulose membrane CN140) was purchased from Sartorius Stedim Biotech GmbH (Goettingen, Germany). The sample pad (glass fiber membrane SB08), absorbent pad (SC2), adhesive backing card, and stacking pads composed of polyester (SP1 (0.5 mm thick) and SP2 (0.1 mm thick)), cellulose (SC1 (1 mm thick) and SC2 (1 mm thick)), and glass fiber (SG1 (0.6 mm thick) and SG2 (0.2 mm thick)) were purchased from ShangHai GoldBio Co. Ltd. (Shanghai, China). The T- and C-lines on the test pad were prepared on the membranes using a Lateral Flow Reagent Dispenser (Claremont BioSolutions, Upland, CA) to dispense the polyclonal anti-Protein A antibody and goat anti-mouse IgG antibody, respectively. Various stacking pads were characterized using scanning electron microscopy (SEM; JSM-6700F, JEOL, Tokyo, Japan). The assembled assay was cut into individual strips of 4 mm in width using a Rapid Test Cutter ZQ2000 (Shanghai kinbio Tech Co., Ltd, China), enabling the platform to be loaded in a commercially available plastic cassette.

### Preparation of AuNPs

AuNPs were synthesized using the Turkevich method^51^. Briefly, 30 nm diameter AuNPs were prepared as follows: A 250 mL two-neck round bottom flask was filled with 100 mL of ultrapure water and placed on a magnetic stirring heater. After boiling, 1 mL of 1% (w/v) trisodium citrate was added with vigorous stirring. After 10 min, 1.2 mL of 0.1% (w/v) chloroauric acid was added to the solution, which changed the color of the solution from yellow to black and then red. We continued to heat and stir the solution for another 10 min and then cooled the sample in a water-ice bath for 30 min.

### AuNP-anti-Protein A and AuNP-anti-CRP antibody conjugate pad preparation

The AuNP-anti-Protein A and AuNP-anti-CRP conjugates were prepared with the same procedure. 1 mL AuNPs was mixed with 1 μg antibody in 0.01 M amine-free PBS solution at pH 8.4. The anti-Protein A or anti-CRP antibodies attached to the surface of the AuNPs over the 45 min incubation period via multiple interactions, including the ionic attraction between the negatively charged gold and the positively charged antibody, the hydrophobic attraction between the gold surface and the antibody, and dative binding between the gold conducting electrons and amino acid sulfur atoms. The antibody/AuNP binding reaction was terminated by adding 0.1 M tris-buffered saline (TBS) with 0.1% (w/v) Tween 20. To remove any excess antibodies, 0.01 M TBS with 0.1% (w/v) Tween 20 was added at 5-times the volume of the conjugate mixture and centrifuged at 8000 g for 35 min. Upon removal of the supernatant, the final conjugates were reconstituted in 0.01 M TBS containing 2% (w/v) bovine serum albumin (BSA). The conjugates were stored at 4 °C until use. To prepare the conjugate pads for the Protein A and CRP assay, the conjugates were first diluted to 0.060 O.D. in the conjugate buffer (2 mM borate buffer with 5% sucrose). The conjugate pads were soaked in the diluted conjugate solution for 1 min, followed by drying at 37 °C for 5 h.

### Fabrication of the LFIA and sLFIA test strips

Compared with the traditional LFIA strip, which is composed of the sample pad, the conjugate pad, the nitrocellulose membrane-based test pad, and the absorbent pad (Fig. 1(a)), the sLFIA includes the added stacking pad between the conjugate pad and the test pad, as shown in Fig. 1(b). A capture antibody, which recognizes and captures Protein A or CRP on the T-line was dispensed and dried on the test pad. Using a micropipette, 10 μL AuNP-antibody conjugate was loaded on the conjugate pad. To immobilize the capture and control probes on the test pad, the capture probes, anti-Protein A or CRP antibody and control probe, and goat anti-mouse IgG were diluted with buffer (2% trehalose in 0.01 M PBS) to 1.5 mg/mL and 1.0 mg/mL, respectively, using the Lateral Flow Reagent Dispenser and kept at 37 °C for 2 h. Next, the test pad (20 mm × 4 mm), absorbent pad (18 mm × 4 mm), conjugate pad (13 mm × 4 mm), and sample pad (15 mm × 4 mm), were sequentially mounted on a plastic adhesive backing pad with 2 mm overlap between each adjacent pads. This structure was termed the “conventional LFIA.” Meanwhile, the sLFIA device was composed of an additional stacking pad (4 mm × 4 mm) with 1 mm overlap between the conjugate pad and the test pad. All parts were assembled according to Fig. 1(b).

### Performance and characterization of various stacking pads

Several different materials, including polyester (SP1 and SP2), cellulose (SC1 and SC2), and glass fiber (SG2 and SG1), were evaluated for their potentials as the stacking pad. To examine the performance of the sLFIA, we compared the results of these six different stacking pads and the conventional LFIA in testing a 10 ng/mL Protein A standard solution loaded at the sample pad (200 µL for each test). The different stacking pads were also characterized with SEM to determine the structure and composition of the materials. Furthermore, we used 1/100 diluted Ponceau S stain solution to observe and verify the fluid stream to determine the stacking effect of the analyte and AuNP conjugated antibodies from the conjugate pad on the stacking pad. Continuous images were captured from 0~60 s for the conventional LFIA and sLFIA tests.

### Quantification of Protein A and CRP

To verify the detection limit of the proposed platform, we compared our sLFIA device with the conventional LFIA method. First we evaluate the sensitivity of the sLFIA device for Protein A and CRP detection using different concentrations of Protein A (250 ng/mL, 125 ng/mL, 100 ng/mL, 10 ng/mL, 5 ng/mL, 1 ng/mL, and blank sample) and CRP (310 ng/mL, 155 ng/mL, 103 ng/mL, 31 ng/mL, 15.5 ng/mL, 3.1 ng/mL, and blank sample) spiked in PBS buffer, respectively. In addition, we also analyzed human serum and synovial fluid samples with various concentrations of CRP using our sLFIA device. Dispense 1 μL of serum or synovial fluid sample into 99 μL of PBS buffer and mix by gentle inversion. Load 120 μL of 1/100-fold diluted serum or synovial fluid sample into the sample well of LFIA device. After loading each sample and waiting for 20 min, the color intensities at the T- and C-lines of the strips were captured using a CCD camera and analyzed using ImageJ software^52^ (National Institutes of Health, Bethesda, MD).
